# Round the Hospitals

**Published:** 1921-08-20

**Authors:** 


### ROUND THE HOSPITALS.

In connection with our article on the Territorial Force Nursing Service, published in our issue of July 30, we have been requested to give the following additional information relating to the functions of the Principal Matrons. The Principal Matrons, who are matrons of leading training-schools, are responsible for enrolling the required number of members in their respective ranks and of keeping their nominal rolls up to date. These nominal rolls are forwarded annually to the Matron-in-Chief of the Service. In effect, not only are the Principal Matrons of the T.F.N.S. consulting officers, but they are recruiting officers also. Intending candidates for the service can obtain all necessary particulars relating to it by applying to the Matron-in-Chief or to the Principal Matron of their district. It is important to realise that good recruits are needed.

The admirable scheme undertaken by the South African Trained Nurses' Society for establishing a memorial to South African nurses who fell during the war has reached a stage when unforeseen difficulties have begun to make themselves felt. The main object of the memorial fund is to establish a home for disabled and superannuated nurses. This it was proposed to build on the coast, but the Transvaal nurses have been specially enthusiastic in raising funds and are now pressing for a memorial home at Johannesburg. It would be excellent

**ROUND THE HOSPITALS**—(*continued*).

to have a home in each province, but such a course is financially impossible, and, unless the South African nurses can agree to unite their forces, we fear there is very little chance of a memorial home worthy of its great purpose being built at all.

Our readers will be amused to hear of the curious means adopted by South African doctors and nurses to bring their names before the public. We learn that the following notices are common examples: "Death. Passed away at Begonia Villas, Mrs. J. S., wife of Mr. J. S. Thanks to Nurse B." "Birth. At Nasturtium Lodge, on the 5th inst., Mrs. T. W., of a son. Thanks to Dr. C." The South African Trained Nurses' Association is attempting to stop the custom, and begs all nurses to discountenance such a use of their names.

The authorities of St. Bartholomew's Hospital have decided on an increase in their rates of pay to probationers, which are in future to be £20, £25, £30, and £40 respectively for each of the four years of their training. This scale is rather higher than that recommended by the College of Nursing.

Miss M. Deane, who has been Matron at the East Suffolk and Ipswich Hospital for the past twenty-two years, is retiring, and the Board of Management has appointed Miss Katharine Violet S. Merriman to the office. Many changes have taken place in the East Suffolk Hospital since the day when Miss Deane was appointed. It was then an institution of less than 100 beds and its work was maintained by an annual expenditure of £4,827. She is leaving an institution of 300 beds and many special departments which require an income of £45,000 per annum to maintain. Not the least important of the hospital's work has been its nursing service, and Miss Deane has every reason to be proud of the staff of ninety-six nurses and sisters whose training and management have been in her hands. In the newly appointed matron the Board expects with every confidence to find a lady eminently qualified to maintain the reputation of the nursing department of the hospital. Miss Merriman, who is a daughter of Canon Merriman, of Hulme, Manchester, received her training at King's College Hospital, London, where she was afterwards retained as a sister. During the war she was matron of a large military hospital with the British Expeditionary Force, and, later, assistant manager of the canteen department of Sir W. G. Armstrong, Whitworth and Co. Miss Merriman's practical work includes service in all the important departments of a large general hospital, together with the training of probationers. In her new appointment she should find full scope for her initiative and administrative faculties.

Prize-day at the East Suffolk and Ipswich Hospital brought together a large gathering, at which unusually interesting reports were furnished by the Matron, Miss Deane, and by the Examiner, Mr. Russell Howard, C.B.E., F.R.C.S. All the nurses but one passed the examinations, and Mr. Howard expressed himself particularly pleased with the quickness and intelligence displayed in the *viva voce*, which, in his opinion, forms a better test of a nurse than written papers. The medallists were Nurse Shepherd (Gold); Nurse Meyes (Silver). Dr. Sinclair concluded with a warm eulogy on Miss Deane, who is retiring from office after many years' work and is greatly beloved by all her colleagues.

The Evelina Hospital for Children petitions for contributions from any who bear the name Evelina or any of its numerous derivatives, such as Eveline, Evelyn, Eva, Eve, Lena, Ina, Ena, etc., to form a Fund to be known as the Evelina Name Fund. We are sure that our readers will let this be known among their friends. Gifts of clothing, articles of food, toys and old linen will be welcomed. The hospital is situated in one of the poorest districts in London, and is sorely pressed to provide for its yearly *clientèle* of 1,000 in the wards and 20,000 out-patients.

The Samaritan Fund of St. Thomas's Hospital aptly illustrates the irresistible power of loving-kindness and brains working together for the betterment of social conditions. To read the annual report is to be cheered and inspired. Surely, the subscriber must reflect, here and thus money and service freely bestowed are put out to good interest. Among the many activities of the Fund we note convalescent treatment afforded to 350 patients from the wards and 732 out-patients; surgical appliances provided for 2,101 and dentures for 198 patients, much of the cost being contributed by patients or repaid after loans from the Fund. The tuberculosis department deals with a bewildering amount of home visiting and reports, yet never loses sight of the individual. Here is radical preventive and curative work, grounded on thorough methods and for ever progressing towards sounder ideals. The speech clinic deals with many cases of incipient feeble-mindedness and sets the backward child on the road to normal citizenship. The maternity and child-welfare department is perhaps the best in London, and has already appreciably raised the standard of home life in its neighbourhood. More money, more convalescent aid, more voluntary workers willing to take serious training are needed There can be few higher privileges than to be permitted a share in work thus conceived and carried through.

The Okehampton Guardians, having appointed Nurse Hinton to be infirmary nurse at their institution, were informed that she was not at liberty to accept that post, as she was under a written agreement to the Devon Nursing Association for two years after obtaining her certificate, of which period she had completed only three months. The Central Committee stated her training had cost £54 in actual money subscribed for the sole purpose of training district nurses. We understand that Nurse Hinton persists in accepting service under the Guardians and has agreed to pay the penalty of £20 and a further sum of £16. It is an unfortunate change of purpose, and our sympathies are strongly with the

Devon Nursing Association. The difficulties, already very great, in the way of training district nurses would become insuperable if all nurses felt themselves at liberty to take other engagements as soon as their training was complete.

An inquest has been held at Woolwich on the bodies of the two babies, aged respectively twenty-two and eighteen months, who died at the M. A. B. Goldie Leigh Homes at Plumstead from zymotic enteritis. In neither case was the doctor sent for till after death, and a searching inquiry was made into all the circumstances. After hearing the evidence of the matron, the assistant matron, the senior nurse, and the house-mother, the conclusion reached by the coroner was that the doctor could have done very little and that no blame attaches to any of the hospital authorities. "They did all they could."

The much-discussed problem of the 2,000,000 women for whom, to judge by statistics, there can be no hope of marriage is closely connected with hospital ideals. So far as we know, there are no available figures estimating what proportion of nurses marry as contrasted with women of other occupations. The marriage rate certainly appears high, to judge by the losses to the profession which must be assigned to this cause. But the important point about nursing is that it does to a very marked extent supply a substitute for marriage. The unmarried woman is often a little hard, and more than a little dissatisfied with life. Her highest instincts, the protective and altruistic desires implanted by nature for the nurture of the new race, are unsatisfied, and when these are starved women miss more even than they know. In nursing these instincts are abundantly brought into play. Though often weary and sometimes out of heart, the nurse is conscious in her own heart of fulfilling her destiny, and is as unlike the typical old maid of fiction as can well be imagined.

The Royal Surrey County Hospital at Guildford has received a splendid gift of £10,000 from the Surrey branch of the British Red Cross Society to build an extension, which is sorely needed, to the nurses' quarters. This is to be the war memorial for the county, and it has been wisely decided to take the nurses' quarters in hand first, instead of building new wards before accommodation has been provided for the nursing staff engaged in them.

The Examination of the Central Midwives Board for Scotland on August 1 and 2 was held simultaneously in Edinburgh, Glasgow, Dundee, and Aberdeen. Out of 106 candidates who appeared for the Examination ninety-two passed. Twenty-eight of these were trained at the Royal Maternity Hospital, Glasgow; twenty-three at the Royal Maternity Hospital, Edinburgh; four at the Maternity Hospital, Aberdeen; twelve at the Maternity Hospital, Dundee; five at the Queen Victoria Jubilee Institute, Edinburgh; ten at the Cottage Nurses' Training Home, Govan, Glasgow; and the remainder at various recognised institutions.

Owing to the great demand for nurses throughout the country, and especially in Government hospitals, the United States Public Health Service has decided to establish training-schools in such of its hospitals as may be fitted for the work. Schools will be opened in the hospital at Fox Hills, Staten Island, New York, and at Fort McHenry, near Baltimore, and in other hospitals as conditions permit.

In a recent number of the *American Journal of Nursing* we learn in the report of Dr. Lyon of the terrible plight of Austrian nurses in Vienna hospitals. With the prevailing prices the entire wage for at least ten months is required to purchase one pair of shoes of the poorest quality. The nurses are housed and fed by the hospital on the small ration which is all that is obtainable. Consumption is rife among them. The contrast with conditions in the United States, where private nurses are said often to charge eight dollars a day, is pointed out by the Medical Director of the Red Cross Relief Unit in Austria. The latest news is that nurses and doctors alike have threatened to strike unless their conditions of service be improved.

A revolutionary suggestion comes from Irish nurses in favour of replacing the orthodox nurse's apron by a white coat or overall, which could be removed and left in the ward when the nurse is not on duty. A grey coat-frock is suggested for indoor uniform, with removable long sleeves. For outdoor uniform, a grey coat and skirt with hat or storm-cap in bad weather is preferred, with grey or black shoes and grey stockings. The General Nursing Council will not lack a good variety to choose from in finally deciding on the registered uniform. But they cannot please everybody.

There has been a cordial response to the Lord Provost's appeal at Glasgow for funds to provide a home for aged and retired nurses. Nearly £16,000 has now been contributed, including £2,609 from the sale of work in the Glasgow Western Infirmary; £1,245 from the garden fête held in the grounds of the County Hospital, Motherwell, organised by the hospital, public health, and district nursing staffs of Lanarkshire; £1,214 from the sale of work at the Glasgow Royal Infirmary, £430 from the sale of work held by the matrons and nursing staffs of the Greenock Hospital and Infirmary and the Gateside Fever Hospital, £400 by the sale of work at the Oakbank Hospital, £258 collected by Queen's nurses in Scotland, £100 contributed by the nursing staff of the Royal Infirmary, Edinburgh, £80 from the nursing staff of the Glasgow Co-operation of Trained Nurses, besides other generous nursing efforts. The Committee aim at a total of £28,000.

The proposed memorial to Sister Edith Johncock, for sixteen years at the Nazareth Hospital, still comes short of the £400 needed to endow and name a bed in that institution. Subscriptions will be gratefully received by Dr. Lechmere Taylor, 36 George Square, Edinburgh.

